# The effects of habitual resistance exercise training on cerebrovascular responses to lower body dynamic resistance exercise: A cross‐sectional study

**DOI:** 10.1113/EP091707

**Published:** 2024-06-18

**Authors:** Stephanie Korad, Toby Mündel, Blake G. Perry

**Affiliations:** ^1^ School of Health Sciences Massey University Wellington New Zealand; ^2^ School of Sport, Exercise and Nutrition Massey University Palmerston North New Zealand; ^3^ Department of Kinesiology Brock University St Catharines Ontario Canada

**Keywords:** blood pressure, middle cerebral artery blood velocity, resistance exercise

## Abstract

Dynamic resistance exercise (RE) produces sinusoidal fluctuations in blood pressure with simultaneous fluctuations in middle cerebral artery blood velocity (MCAv). Some evidence indicates that RE may alter cerebrovascular function. This study aimed to examine the effects of habitual RE training on the within‐RE cerebrovascular responses. RE‐trained (*n* = 15, Female = 4) and healthy untrained individuals (*n* = 15, Female = 12) completed four sets of 10 paced repetitions (15 repetitions per minute) of unilateral leg extension exercise at 60% of predicted 1 repetition maximum. Beat‐to‐beat blood pressure, MCAv and end‐tidal carbon dioxide were measured throughout. Zenith, nadir and zenith‐to‐nadir difference in mean arterial blood pressure (MAP) and mean MCAv (MCAv_mean_) for each repetition were averaged across each set. Two‐way ANOVA was used to analyse dependent variables (training × sets), Bonferroni corrected *t*‐tests were used for *post hoc* pairwise comparisons. Group age (26 ± 7 trained vs. 25 ± 6 years untrained, *P* = 0.683) and weight (78 ± 15 vs. 71 ± 15 kg, *P* = 0.683) were not different. During exercise average MAP was greater for the RE‐trained group in sets 2, 3 and 4 (e.g., set 4: 101 ± 11 vs. 92 ± 7 mmHg for RE trained and untrained, respectively, *post hoc* tests all *P* = < 0.012). Zenith MAP and zenith‐to‐nadir MAP difference demonstrated a training effect (*P *< 0.039). Average MCAv_mean_ and MCAv_mean_ zenith‐to‐nadir difference was not different between groups (interaction effect *P* = 0.166 and *P* = 0.459, respectively). Despite RE‐trained individuals demonstrating greater fluctuations in MAP during RE compared to untrained, there were no differences in MCAv_mean_. Regular RE may lead to vascular adaptations that stabilise MCAv during RE.

## Abstract (highlights)

- **What is the central question of this study?**
  Resistance exercise produces vascular adaptations within the central arteries: do these adaptations extend to the cerebral circulation? What are the effects of habitual resistance exercise training on the within‐resistance exercise cerebrovascular responses?
- **What is the main finding and its importance?**
  During dynamic resistance‐exercise, resistance‐trained individuals demonstrated greater mean arterial pressure, but mean middle cerebral artery blood velocity did not differ from untrained individuals, indicating improved cerebral blood flow regulation. These findings suggest that functional cerebrovascular adaptations may have occurred during repetitive exposure to fluctuations in blood pressure.

## 1 INTRODUCTION

Resistance exercise (RE) is a popular form of exercise due to the many associated physiological benefits, such as increased muscle mass and strength (Deschenes & Kraemer, 2002), reduced fat mass (Lopez et al., 2022), neuroprotection (Yarrow et al., 2010) and improved mental well‐being (O'Connor et al., 2010). The physiological benefits of RE extend to clinical populations, with RE used as treatment for cardiovascular disease (Strasser & Schobersberger, 2011), diabetes mellitus (Evans et al., 2019) and sarcopenia (Seguin & Nelson, 2003). However, RE induces substantial perturbations in arterial blood pressure (ABP), with recordings of systolic (SBP) and diastolic (DBP) blood pressures reaching 480 and 350 mmHg, respectively, during high intensity dynamic RE (MacDougall et al., 1985). Conversely, acute hypotension, sufficient to produce syncope has been observed following the cessation of intense RE (Moralez et al., 2012; Perry et al., 2014; Romero & Cooke, 2007); however, syncope typically only occurs following maximal efforts with a concurrent Valsalva manoeuvre (Compton et al., 1973). Despite the small potential for harm, RE should be encouraged for overall physical (Kraemer et al., 1988) and psychological well‐being (O'Connor et al., 2010).

During changes in ABP the vasculature of the brain responds by modifying vessel radius to minimise variations in cerebral blood flow (CBF), a process termed cerebral autoregulation. Whilst cerebral autoregulation is a potent regulator of CBF, as with any physiological system there is a delay between stimulus and response, with an ∼5 s lag between the change in perfusion pressure and subsequent cerebrovascular response (Zhang et al., 1998). The inherent lag in the cerebral autoregulatory process generates a high pass filter, that is, higher frequency oscillations in blood pressure are translated to the cerebral circulation largely unbuffered (Zhang et al., 2002). Such a situation exists during dynamic RE where the rapid sinusoidal fluctuations in ABP are reflected in concurrent changes in middle cerebral artery blood velocity (MCAv) (Edwards et al., 2002; Perry et al., 2014; Romero & Cooke, 2007). Furthermore, blood pressure increases in subsequent sets of the same RE (Libardi et al., 2017). Indeed, several studies have suggested that the intermittent ABP extremes during high intensity RE underpins the reduction in central arterial compliance following a single bout of RE (DeVan et al., 2005) and at rest in RE‐trained individuals (Miyachi, 2013; Miyachi et al., 2004), with increased arterial stiffness associated with elevated cardiovascular disease risk (Mattace‐Raso et al., 2006; Mitchell et al., 2010).

As the ABP profile during RE is translated to the cerebral circulation, it is unclear if habitual RE training and the associated repetitive exposure to fluctuating ABP illicit vasculature adaptations within the brain as occurs in the central arteries. We have previously reported that there was no difference between cerebral autoregulatory capacity between sedentary and RE‐trained individuals, with only a trend for lower phase in the RE‐trained group at a frequency of 0.05 Hz (Perry et al., 2019). Further analysis of our data revealed that RE‐trained individuals did not exhibit the hysteresis pattern of cerebral autoregulation, with only sedentary and endurance trained individuals exhibiting greater cerebral autoregulatory capacity during hypertensive challenges (Roy et al., 2022). Thomas et al. (2021), in a randomised and cross over study design, investigated the effects of endurance exercise and RE on cerebrovascular function and reported that RE increases cerebrovascular resistance and decreases pulsatility index (PI) at rest. However, Thomas et al. (2021) did not quantify the effect of habitual exercise on within‐exercise RE responses. These findings collectively indicate that habitual exercise may subtly modify cerebrovascular function, yet the impact of habitual RE training on the within‐RE cerebrovascular responses has yet to be determined. The aim of this study was to assess the impact of dynamic RE on cerebrovascular responses in RE‐trained and untrained individuals. We hypothesised that RE‐trained individuals would exhibit smaller fluctuations in MCAv during exercise compared to their untrained counterparts.

## 2 METHODS

### 2.1 Ethics and informed consent

All participants were informed of the experimental procedures and aware of the purpose of this study, as well as the potential risks associated with participating. All participants provided written informed consent prior to taking part in the research. The study was approved by the Massey University Human Ethics Committee (SOA 21/22) and was in agreement with the latest version of the *Declaration of Helsinki* apart from registration in a database. This study was part of a larger study investigating cerebrovascular responses to RE. However, all data presented herein were collected independently and not influenced by the additional aims and outcomes.

### 2.2 Participants

An a priori power analysis (G*Power version 3.1.9.4; Heinrich Heine University Düsseldorf, Düsseldorf, Germany) was conducted using data from Edwards et al. (2002) and Moralez et al. (2012) with similar interventions (dynamic resistance exercise), design and outcome measures (i.e., MCAv and MAP). Based on conventional α (0.05) and β (0.80) values, a minimum of 24 participants (*n* = 12 per training group) was required. A total of 30 participants (female = 16) were recruited for this study (pooled mean ± SD: age, 26 ± 6 years, height 175 ± 10 cm, weight 74 ± 15 kg, body mass index 24 ± 5 kg/m^2^), with 15 participants in each group (see Table 1 for training group anthropometric data). All participants were healthy and free of any medical conditions, were not taking any form of medication other than oral contraception (RE‐trained *n* = 1, untrained *n* = 3), or an intrauterine device (untrained = 1), were non‐smokers, and had no history or symptoms of cardiovascular, pulmonary, metabolic or neurological disease. Menstrual cycle phase was self‐reported by female participants with all visits occurring during the early follicular phase (low oestrogen and progesterone) and during the placebo phase for those using oral contraceptives. Korad et al. (2022) and Favre & Serrador (2019) have previously reported no differences in functional cerebrovascular responses to acute changes in MAP and cerebral autoregulation between menstrual cycle phases. The participants also self‐reported their habitual exercise regimen to be assigned to one of the following training groups: resistance‐trained individuals: classified as having completed any modality (Olympic, bodybuilding, powerlifting) of RE training for ≥30 min, ≥3 times per week for ≥6 months prior to the experiment; and healthy sedentary: ≤1 dedicated exercise session per week for ≥6 months prior to the experiment. This does not include regular physical activity, for example, activities that would be classified as low intensity such as walking, gardening, low‐intensity cycling (commuting) and general household chores. If the participant engaged in rowing, they were excluded from the study as rowing produces similar blood pressure fluctuations to RE (Pott et al., 1997).

**TABLE 1:** Participants anthropometric and strength measurements.

Variable	RE‐trained	Untrained	*P*
Sex (male:female)	(11:4)	(3:12)	N/A
Age (years)	26 ± 7	25 ± 6	0.683
Height (m)	1.77 ± 0.09	1.72 ± 0.09	0.167
Weight (kg)	78 ± 15	71 ± 15	0.683
BMI (kg/m^2^)	25 ± 4	24 ± 6	0.809
Leg extension predicted 1RM (kg)	76 ± 19	52 ± 15	**<0.001**
Leg extension 60% of 1RM (kg)	44 ± 12	30 ± 8	**<0.001**
RE experience (months)	49 ± 45	—	—

*Note*: Data are presented as means ± SD. Abbreviations: 1RM, one repetition maximum; BMI, body mass index; RE, resistance exercise; RE‐trained, resistance exercise trained.

### 2.3 Study design

All participants visited the temperature‐controlled laboratory twice, once initially for familiarisation and lastly for the experimental session. A full explanation and demonstration of the risks of participation, and equipment and procedures utilised in the experiment were given during the familiarisation session. Upon providing consent, the middle cerebral artery (MCA) contralateral to the exercising limb was insonated for the measurement of MCAv as described below. In addition, the participant's unilateral leg extension one repetition maximum (1RM, dominant leg) was estimated using the Brzycki (1993) equation as: Weight/[1.0278 − (0.0278 × Number of repetitions)]. The working intensity for the trial, 60% of the 1RM (60%1RM), was calculated. The participant also practised executing the leg extension at 60%1RM whilst maintaining the requested pacing and breathing pattern outlined below.

### 2.4 Experimental protocol 

**FIGURE 1:**
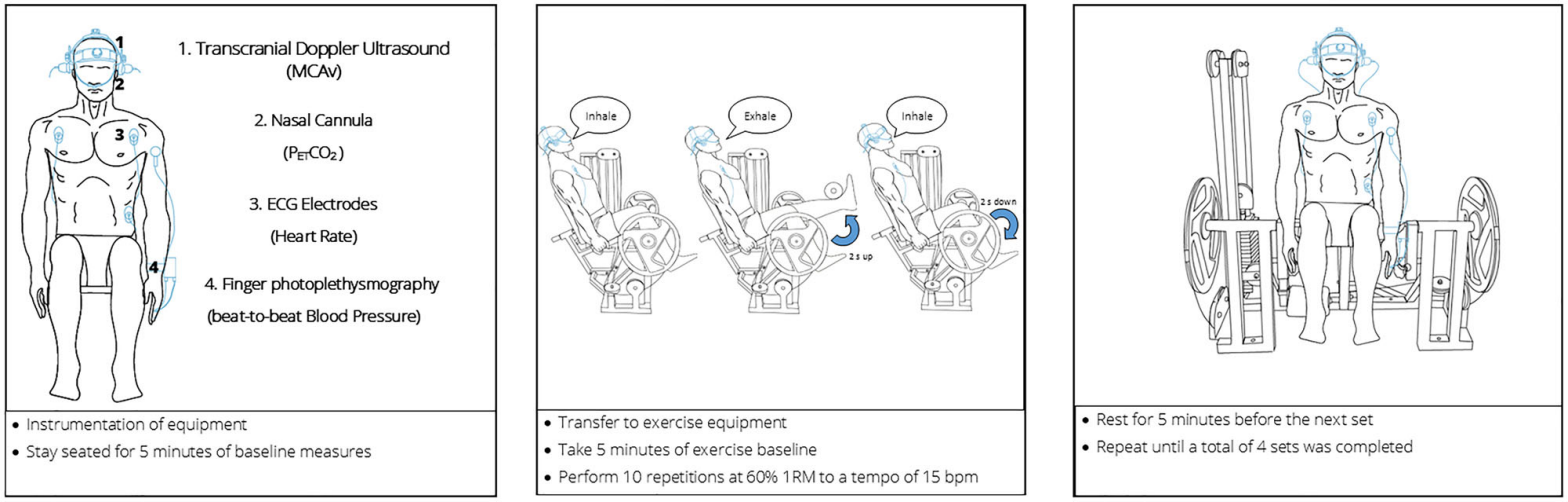
Experimental protocol. The exercise sets consisted of 10 repetitions of unilateral leg extensions. Haemodynamic variables (MCAv, blood pressure and heart rate) and partial pressure of end‐tidal carbon dioxide ($P_{\mathrm{ETC}O_{2}}$) were measured throughout.

The familiarisation occurred >1 week before the trial. Participants arrived at the laboratory having refrained from caffeinated beverages for 12 h, vigorous exercise and alcohol consumption for ≥24 h prior to testing. The participants were also instructed to consume 500 mL of water the night before and 500 mL approximately 4 h before the experiment to ensure euhydration (urine specific gravity, USG < 1.020). The experimental overview is highlighted in Figure 1. 

On arrival, the participant was asked to provide a urine sample for USG analysis. The participant was then seated on a chair for instrumentation. Once instrumented the participant rested quietly for 20 min for initial baseline recordings. Upon completion of baseline measures the participant was then transferred to the leg extension machine. Baseline values were continued for another 5 min, and immediately preceding each exercise set thereafter. During the exercise phase, the participant performed 10 repetitions of unilateral leg extensions at 60%1RM to a tempo of 15 bpm, which equates to a repetition cycle length of 4 s (2 s per concentric and eccentric phase). The breathing sequence was set to match the tempo of the exercise, with exhalation during the concentric phase (2 s), and inhalation during the eccentric phase (2 s). As such, all participants avoided the Valsalva manoeuvre during RE. The participant then rested for 5 min whilst baseline measures were taken, before repeating the sequence again until a total of four sets of 10 repetitions were completed. Each participant was reminded of the breathing technique prior to each set, with the breathing and repetition timing aided by a metronome. The following criteria was used to ensure that the Valsalva manoeuvre was not performed:

1. No large and acute elevations in blood pressure, beyond what would be expected for the intensity of exercise as gauged by previous repetitions, were noted. Previous studies reported that when the Valsalva manoeuvre is recruited during resistance exercise, MAP (but not necessarily MCAv) is acutely elevated within the repetition (Perry et al., 2020, 2014).
2. Participants produced an expected capnograph that aligned with the paced breathing of the repetitions.
3. Participants were reminded of the exercise and breathing requirements for each set prior to completion.

### 2.5 Systemic haemodynamics

Heart rate (HR) was measured using a three‐lead electrocardiogram (ECG; ADInstruments, Bella Vista, Australia). Non‐invasive beat‐to‐beat ABP was measured by finger photoplethysmography (Finapres Medical Systems, Enschede, The Netherlands). The cuff was placed on the middle phalanx of either the middle finger or the index finger on the non‐dominant hand. The cuff was referenced to the level of the heart using the height correction unit. During the familiarisation the participants were instructed on the ways they can hold the leg extension grip without compromising the measurements of the Finometer. The middle panel of the schematic representation in Figure 1 obscures the left hand, which for the purposes of this schematic depiction only, has the Finometer attached. The Finometer was placed on the non‐dominant hand, and the contralateral hand was used to grip the leg‐extension machine. The non‐dominant hand rested on the participant's lap when they performed the exercise to prevent any movement or pressure artefacts in the blood pressure trace. Blood pressure values were checked against an automated sphygmomanometer (Suresigns VM4, Philips Medical Systems, Best, The Netherlands) during baseline and 2 min following each exercise bout and corrected when necessary.

### 2.6 Middle cerebral artery blood velocity

Blood velocity in the contralateral middle cerebral artery (MCA) to the exercising leg was measured using transcranial Doppler (TCD) ultrasonography (Doppler‐Box X, DWL, Compumedics, Singen, Germany). The contralateral MCA was selected as the motor tracts are decussate. Thus, it is possible that neurovascular coupling, the matching of cerebral perfusion with local neuronal activity, would mediate a larger increase in the contralateral MCAv compared to the ipsilateral side, as seen in static handgrip exercise (Braz et al., 2014). Blood velocity in the M1 segment of the MCA was measured using a 2 MHz probe, fixed in position with an adjustable headband. The probe was fixed over the temporal window, above the zygomatic arch, using search techniques described elsewhere (Aaslid et al., 1989; Willie et al., 2011). Ultrasound gel (Tensive, Parker Laboratory, Fairfield, NY, USA) was placed between the transducer probe and the skin to ascertain the highest quality image. The average depth of the insonated MCA in the current study was 54 ± 4 mm in alignment with Bathala et al. (2013).

### 2.7 Partial pressure of end‐tidal carbon dioxide

The partial pressure of end‐tidal carbon dioxide ($P_{\mathrm{ETC}O_{2}}$) was measured using an online gas analyser (ML206 Gas Analyser, ADInstruments) and was collected throughout using a nasal cannula. The gas analyser was calibrated to a known gas concentration before each experiment.

### 2.8 Urine analysis

Hydration status has been reported to influence cerebrovascular regulation (Moralez et al., 2012; Perry et al., 2016), and therefore USG was used to confirm hydration status before each experiment using a handheld refractometer (Atago Co., Ltd, Tokyo, Japan). All participants were instructed to consume 500 mL of water the night before and 500 mL approximately 4 h before the experiment. Approximately 30 min before the commencement of the experiment, USG was measured to confirm euhydration (mean ± SD 1.010 ± 0.007). If the participant did not meet the USG requirement, ∼500 mL of water was given to the participant and USG was retaken 30 min after the consumption water, until a value of <1.020 was returned.

### 2.9 Data acquisition

All data were collected continuously using an analogue to digital converter (PowerLab, ADInstruments) interfaced with a computer and then analysed using LabChart software (v.8.1.13 ADInstruments).

### 2.10 Data analysis

#### 2.10.1 Dependent measures

Mean MCAv (MCAv_mean_) was calculated using the mean waveform of the raw MCAv trace and mean arterial blood pressure (MAP) was calculated using the equation 1/3 SBP + 2/3DBP. The cerebrovascular conductance index (CVCi) was calculated using the equation CVCi = MCAv_mean_/MAP. The Gosling pulsatility index (PI) for the MCA was calculated as: SMCAv − DMCAv/MCAv_mean_ (Gosling & King, 1974), where SMCAv represents the maximum blood velocity in the MCA during systole and DMCAv the minimum blood velocity in the MCA during diastole. Additionally, pulse pressure (PP) was calculated as SBP − DBP. Given the sinusoidal haemodynamic profile during RE, the zenith and nadir MCAv_mean_ and MAP values were identified for each repetition and the average values were calculated for each set. Additionally, zenith‐to‐nadir MCAv_mean_ and MAP values were calculated as the zenith MCAv_mean_ value − the nadir MCAv_mean_ value and zenith MAP value − the nadir MAP during each repetition, respectively. The average zenith‐to‐nadir values for MCAv_mean_ and MAP were calculated for each set.

### 2.11 Statistical analysis

All data were analysed using SPSS Statistics software version 28 (IBM Corp., Armonk, NY, USA). Statistical significance was set at *P ≤* 0.05. An unpaired Student's *t*‐test was performed to compare training anthropometric, 1RM and 60%1RM data. A two‐way mixed ANOVA was performed to analyse baseline measures (training × baselines, 2 × 5) and dependent variables of interest during dynamic RE (training × sets, 2 × 5 when initial baseline is included as with mean data, and 2 × 4 when within exercise only data are analysed, for example, Zenith MCAv_mean_). *t*‐Tests were used for *post hoc* comparisons and a Bonferroni correction factor was used when necessary. Partial eta square (partial η^2^) is reported for the training by set interaction only, with large effect sizes identified as >0.1379, medium 0.0588–0.1379, and small <0.0099 (Cohen, 2013). All data are displayed as the mean ± SD. 

## 3 RESULTS

Participants’ anthropometric and exercise measurements are presented in Table 1. There were no significant differences in the anthropometric measurements between the RE‐trained and untrained; however, RE‐trained had a greater predicted 1RM and 60% of 1RM versus their untrained counterparts (see Table 1 for *P* values). The RE‐trained group trained for 49 ± 45 months, ranging from 6 to 144 months of continuous resistance training.

### 3.1 Baseline measurements

Baseline measures for the initial baseline immediately following instrumentation and baseline prior to each set are shown in Table 2. There were no significant main effects of training or training by set interaction for any baseline variables between groups. However, except for heart rate, there was a main effect of set for all variables, whereby both training groups demonstrated equal ‘drift’ in the baseline variables.

**TABLE 2:** Resistance‐trained versus untrained baseline cerebrovascular and cardiovascular measures.

		Baseline period					*P*			
Variable	Training group	Initial	Prior to set 1	Prior to set 2	Prior to set 3	Prior to set 4	Training	Set	Interaction	Partial ƞ^2^
**MCAv_mean_ (cm s^−1^)**	RE‐trained	65 ± 8	68 ± 11	67 ± 10	66 ± 10 ^b^	65 ± 10 ^b^	0.274	**<0.001**	0.866	0.011
	Untrained	69 ± 11	72 ± 11	71 ± 11	69 ± 11 ^b^	69 ± 11 ^b^				
**MAP (mmHg)**	RE‐trained	82 ± 10	86 ± 12 ^a^	87 ± 13 ^a^	87 ± 11 ^a^	90 ± 12 ^a^	0.628	**<0.001**	0.945	0.007
	Untrained	81 ± 6	84 ± 9 ^a^	86 ± 8 ^a^	85 ± 8 ^a^	88 ± 8 ^a^				
**CVCi (cm s^−1^ mmHg^−1^)**	RE‐trained	0.81 ± 0.15	0.80 ± 0.17	0.78 ± 0.17	0.77 ± 0.18 ^a b^	0.76 ± 0.20 ^a^	0.366	**<0.001**	0.881	0.010
	Untrained	0.86 ± 0.14	0.86 ± 0.13	0.783 ± 0.14	0.81 ± 0.12 ^a b^	0.80 ± 0.14 ^a^				
**PP (mmHg)**	RE‐trained	55 ± 10	54 ± 10	56 ± 11	54 ± 10 ^c^	52 ± 11 ^c^	0.501	**0.026**	0.907	0.009
	Untrained	57 ± 13	57 ± 12	58 ± 14	57 ± 13 ^c^	56 ± 14 ^c^				
**PI**	RE‐trained	0.84 ± 0.13	0.81 ± 0.14 ^a^	0.84 ± 0.14 ^b^	0.82 ± 0.14	0.81 ± 0.15	0.941	**0.006**	0.645	0.022
	Untrained	0.83 ± 0.18	0.79 ± 0.16 ^a^	0.83 ± 0.16 ^b^	0.84 ± 0.20	0.81 ± 0.16				
$P_{\mathrm{ETC}O_{2}}$ **(mmHg)**	RE‐trained	38 ± 5	39 ± 5	38 ± 4	38 ± 4	38 ± 4 ^b^	0.162	**0.032**	0.644	0.022
	Untrained	36 ± 4	37 ± 4	36 ± 4	36 ± 4	36 ± 4 ^b^				
**HR (bpm)**	RE‐trained	73 ± 14	73 ± 14	71 ± 14	70 ± 14	72 ± 14	0.821	0.344	0.070	0.074
	Untrained	70 ± 16	70 ± 14	72 ± 15	70 ± 15	72 ± 15				

*Note*: Data are presented as means ± SD. Resistance‐trained, *n* = 15; untrained, *n* = 15. ^a^Different from initial. ^b^Different from set 1. ^c^Different from set 2. Abbreviations: CVCi, cerebrovascular conductance index; HR, heart rate; MAP, mean arterial pressure; MCAv_mean_, middle cerebral artery blood velocity mean; $P_{\mathrm{ETC}O_{2}}$, end‐tidal partial pressure of carbon dioxide; PI, pulsatility index; PV, pulse velocity; RE‐trained, resistance‐trained.

### 3.2 Averaged response to dynamic resistance exercise

**FIGURE 2:**
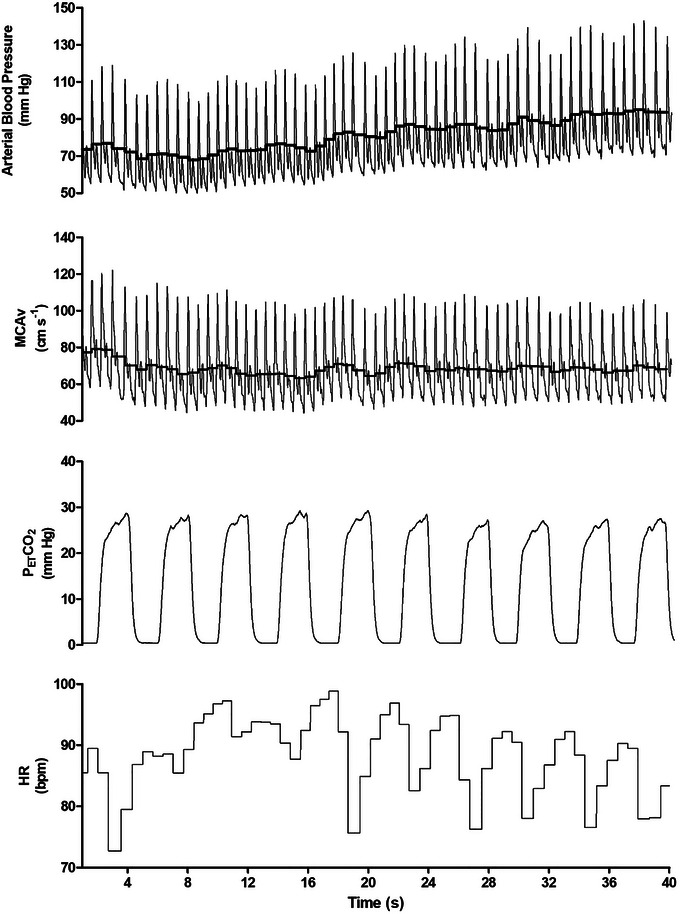
Typical trace of middle cerebral artery blood velocity (MCAv), arterial blood pressure, partial pressure of end tidal carbon dioxide ($P_{\mathrm{ETC}O_{2}}$) and heart rate (HR) during exercise. The thick black line in the MCAv and arterial blood pressure traces represent mean MCAv and mean arterial pressure, respectively. The numbers indicate the rep count with the dotted line denoting the start (concentric phase) of each repetition.

The typical response to dynamic resistance exercise is detailed in Figure 2, with the averaged cerebrovascular and cardiovascular responses within exercise presented in Table 3. A training by set interaction was seen in MAP (*P* = 0.010) and SBP (*P* < 0.001). Post‐hoc tests revealed higher MAP in the RE‐trained group in sets 2, 3 and 4 (all *P* = < 0.012), and in all 4 sets (all P = < 0.001) for SBP (see Table 3 for values). A training by set interaction was demonstrated for DMCAv (*P* < 0.001) (see Table 3 for values), although *post hoc* tests revealed no differences (all *P* > 0.113). A set effect was seen for MCAv_mean_, CVCi, PP and $P_{\mathrm{ETC}O_{2}}$ (all *P* < 0.001), which reflected values decreasing from set 1 to 4 in both groups, whilst MAP and HR had set differences of *P* < 0.001, reflected by increasing values (see Table 3 for values).

**TABLE 3:** Averaged cerebrovascular and cardiovascular within exercise response during dynamic resistance exercise.

		Sets					*P*			
Variable	Training group	Baseline	Set 1	Set 2	Set 3	Set 4	Training	Set	Interaction	Partial ƞ^2^
**MCAv_mean_ (cm s^−1^)**	RE‐trained	65 ± 8	63 ± 11	63 ± 10	61 ± 9 ^a,b,c^	61 ± 9^a,b,c^	0.194	**<0.001**	0.166	0.056
	Untrained	69 ± 11	71 ± 11	67 ± 10	65 ± 8^a,b,c^	64 ± 8^a,b,c^				
**SMCAv_mean_ (cm s^−1^)**	RE‐trained	102 ± 12	98 ± 15	96 ± 15	94 ± 14 ^a,b,c^	94 ± 15^a,b,c^	0.370	**<0.001**	0.533	0.027
	Untrained	106 ± 18	105 ± 17	102 ± 17	98 ± 15 ^a b c^	97 ± 15^a,b,c^				
**DMCAv_mean_ (cm s^−1^)**	RE‐trained	47 ± 6	45 ± 8	50 ± 10	49 ± 10	45 ± 8	0.906	0.293	**<0.001**	0.167
	Untrained	49 ± 9	46 ± 7	45 ± 6	45 ± 6	46 ± 7				
**MAP (mmHg)**	RE‐trained	82 ± 10	96 ± 10 ^a^	99 ± 11* ^a^	100 ± 10* ^a^	101 ± 11* ^a^	**0.022**	**<0.001**	**0.010**	0.110
	Untrained	81 ± 6	90 ± 10 ^a^	89 ± 7 ^a^	91 ± 7 ^a^	92 ± 7 ^a^				
**SBP (mmHg)**	RE‐trained	117 ± 14	144 ± 17* ^a^	145 ± 19* ^a^	148 ± 18* ^a^	149 ± 19* ^a^	**0.002**	**<0.001**	**<0.001**	0.190
	Untrained	116 ± 11	127 ± 15 ^a^	126 ± 11 ^a^	127 ± 13 ^a^	125 ± 12 ^a^				
**DBP (mmHg)**	RE‐trained	64 ± 8	72 ± 10 ^a^	77 ± 10 ^a^	77 ± 10 ^a^	78 ± 10 ^a,c^	0.410	**<0.001**	0.566	0.026
	Untrained	64 ± 6	72 ± 11 ^a^	71 ± 10 ^a^	73 ± 8 ^a^	75 ± 8 ^a,c^				
**CVCi (cm s^−1^ mmHg^−1^)**	RE‐trained	0.81 ± 0.15	0.67 ± 0.13 ^a^	0.65 ± 0.14 ^a^	0.62 ± 0.13^a,b,c^	0.62 ± 0.12^a,b,c^	**0.037**	**<0.001**	0.182	0.054
	Untrained	0.86 ± 0.14	0.79 ± 0.13 ^a^	0.75 ± 0.11 ^a^	0.72 ± 0.10^a,b,c^	0.60 ± 0.11^a,b,c^				
**PP (mmHg)**	RE‐trained	55 ± 10	54 ± 10	51 ± 10	50 ± 10^a,b,c^	49 ± 10^a,b,c^	0.477	**<0.001**	0.708	0.019
	Untrained	57 ± 13	56 ± 12	56 ± 13	53 ± 13^a,b,c^	52 ± 12^a,b,c^				
**PI**	RE‐trained	0.84 ± 0.13	0.86 ± 0.15	0.83 ± 0.15	0.83 ± 0.13	0.82 ± 0.14	0.787	0.676	0.111	0.064
	Untrained	0.83 ± 0.18	0.80 ± 0.16	0.84 ± 0.16	0.82 ± 0.17	0.82 ± 0.15				
$P_{\mathrm{ETC}O_{2}}$ **(mmHg)**	RE‐trained	38 ± 5	36 ± 5 ^a^	36 ± 5 ^a^	35 ± 5 ^a,b^	35 ± 5 ^a,b^	0.282	**<0.001**	0.800	0.014
	Untrained	36 ± 4	35 ± 4 ^a^	34 ± 4 ^a^	33 ± 4 ^a,b^	33 ± 4^a,b^				
**HR (bpm)**	RE‐trained	73 ± 14	91 ± 14 ^a^	92 ± 16 ^a^	93 ± 13 ^a^	93 ± 15 ^a^	0.505	**<0.001**	0.567	0.026
	Untrained	70 ± 16	91 ± 13 ^a^	88 ± 14 ^a^	88 ± 13 ^a^	89 ± 12 ^a^				

*Note*: Data are presented as means ± SD. Resistance‐trained, *n = *15; Untrained, *n* = 15. Despite a significant training by set interaction *post hoc* tests revealed no differences between training groups for DMCAv (*P* ≥ 0.113); however, *post hoc* test revealed significant differences in MAP (all *P* ≤ 0.012) and SBP (all *P* ≤ 0.002), differences denoted with *. ^a^Different from initial baseline. ^b^Different from set 1. ^c^Different from set 2. Abbreviations: CVCi, cerebrovascular conductance index; DMCAv, diastolic middle cerebral artery blood velocity; HR, heart rate; MAP, mean arterial blood pressure; MCAv_mean_, mean middle cerebral artery blood velocity; $P_{\mathrm{ETC}O_{2}}$, end‐tidal partial pressure of carbon dioxide; PI, pulsatility index; PV, pulse velocity; RE‐trained, resistance‐trained; SMCAv, systolic middle cerebral artery blood velocity.

### 3.3 Zenith and nadir response to dynamic resistance exercise

**FIGURE 3:**
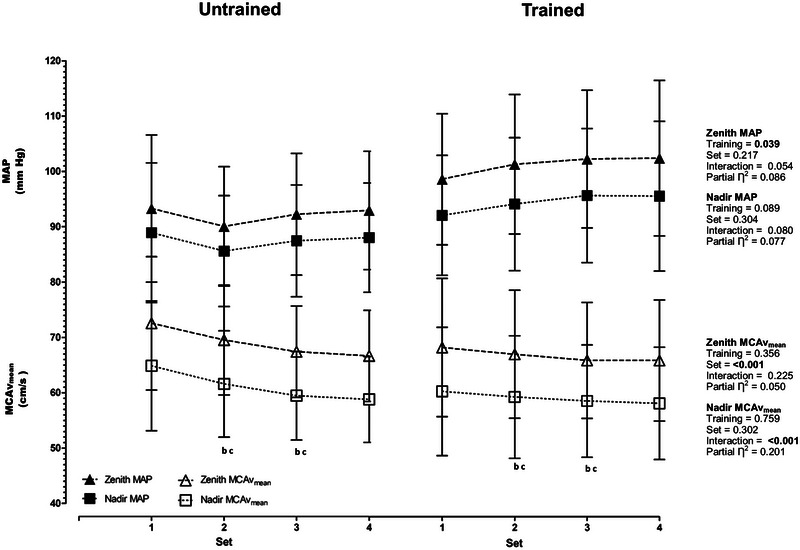
Zenith and nadir mean arterial blood pressure (MAP) and mean middle cerebral artery blood velocity (MCAv_mean_) during dynamic resistance exercise. Data are means ± SD. ^b^Different from set 1. ^c^Different from set 2. Despite a significant training by set interaction, *post hoc* tests revealed no differences between training groups for Nadir MCAv (*P* ≥ 0.293).

Similarly to the averaged responses during exercise, there were set differences for MCAv_mean_, SMCAv, DMCAv and CVCi at zenith MCAv_mean_ and nadir MCAv_mean_ (all *P *< 0.003, see Table 4 for values). The RE‐trained group demonstrated greater SBP at zenith MAP (training effect *P* = 0.006) and MAP nadir (training effect *P* = 0.010, see Table 4 for values), indicating a sustained increase in SBP throughout exercise. Additionally, zenith MAP was greater in the RE‐trained group (training effect *P* = 0.039, see Figure 3).

**TABLE 4:** Zenith and nadir MCAv_mean_, MAP, CVCi and zenith‐to‐nadir of MCAv_mean_ and MAP during dynamic resistance exercise.

		Sets				*P*			
Variable	Training group	Set 1	Set 2	Set 3	Set 4	Training	Set	Interaction	Partial *ƞ* ^2^
**Zenith**									
** SMCAv (cm s^−1^)**	RE‐trained	102 ± 18	99 ± 17	97 ± 14 ^b c^	97 ± 15 ^b,c^	0.550	**<0.001**	0.165	0.058
	Untrained	105 ± 18	102 ± 17	99 ± 15 ^b c^	97 ± 15 ^b,c^				
** DMCAv (cm s^−1^)**	RE‐trained	48 ± 9	47 ± 8	47 ± 7	47 ± 8 ^b^	0.865	**0.003**	0.083	0.076
	Untrained	50 ± 9	48 ± 7	47 ± 6	46 ± 6 ^b^				
** SBP (mmHg)**	RE‐trained	144 ± 20	146 ± 20	148 ± 21	147 ± 22	**0.006**	0.718	0.119	0.067
	Untrained	129 ± 22	124 ± 18	125 ± 18	126 ± 17				
** DBP (mmHg)**	RE‐trained	76 ± 10	79 ± 11	80 ± 10	80 ± 12 ^b^	0.318	**0.017**	0.082	0.076
	Untrained	75 ± 11	73 ± 9	76 ± 10	77 ± 9 ^b^				
** CVCi (cm s^−1^ mmHg^−1^)**	RE‐trained	0.69 ± 0.15	0.66 ± 0.17	0.64 ± 0.15 ^b,c^	0.64 ± 0.16 ^b c^	0.225	**<0.001**	0.489	0.028
	Untrained	0.76 ± 0.14	0.74 ± 14	0.70 ± 0.10 ^b,c^	0.68 ± 0.12 ^b c^				
**Nadir**									
** SMCAv (cm s^−1^)**	RE‐trained	97 ± 17	94 ± 14	92 ± 14 ^b,c^	91 ± 15 ^b,c^	0.690	**<0.001**	0.585	0.023
	Untrained	100 ± 18	97 ± 15	94 ± 15 ^b,c^	92 ± 15 ^b,c^				
** DMCAv (cm s^−1^)**	RE‐trained	43 ± 9	42 ± 8	42 ± 8 ^b^	42 ± 7 ^b^	0.784	**<0.001**	*0.053*	0.087
	Untrained	46 ± 9	43 ± 6	42 ± 5 ^b^	41 ± 5 ^b^				
** SBP (mmHg)**	RE‐trained	136 ± 18	138 ± 19	140 ± 20	141 ± 20	**0.010**	0.651	0.063	0.083
	Untrained	125 ± 19	119 ± 17	120 ± 17	120 ± 16				
** DBP (mmHg)**	RE‐trained	70 ± 10	72 ± 11	74 ± 10	74 ± 12	0.610	*0.052*	0.204	0.053
	Untrained	71 ± 10	69 ± 9	71 ± 9	72 ± 8				
** CVCi (cm s^−1^ mmHg^−1^)**	RE‐trained	0.67 ± 0.15	0.64 ± 0.17	0.62 ± 0.15^b,c^	0.62 ± 0.16 ^b,c^	0.268	**<0.001**	0.446	0.031
	Untrained	0.73 ± 0.13	0.72 ± 14	0.67 ± 0.11 ^b,c^	0.66 ± 0.14 ^b,c^				
**Zenith‐to‐nadir difference**									
MCAv_mean_	RE‐trained	8 ± 3	8 ± 3	7 ± 3	8 ± 2	0.837	0.894	0.459	0.030
	Untrained	8 ± 2	8 ± 2	8 ± 2	8 ± 2				
MAP	RE‐trained	7 ± 2	7 ± 2	7 ± 2	7 ± 2	**0.002**	0.476	0.422	0.033
	Untrained	4 ± 2	4 ± 2	5 ± 2	5 ± 2				

*Note*: Data are presented as means ± SD. Resistance‐trained, *n* = 15; untrained, *n* = 15. ^b^Different from set 1. ^c^Different from set 2. Abbreviations: CVCi, cerebrovascular conductance index; DBP, diastolic blood pressure; DMCAv, diastolic middle cerebral artery blood velocity; MAP, mean arterial blood pressure; MCAv_mean_, mean middle cerebral artery blood velocity; RE‐trained, resistance‐trained; SBP, systolic blood pressure; SMCAv, systolic middle cerebral artery blood velocity.

### 3.4 Zenith‐to‐nadir difference

No significant effect of training was observed in MCAv_mean_ (*P* = 0.837); however, a difference was apparent for MAP (*P* = 0.002), with the RE‐trained showing a significantly greater zenith‐to‐nadir difference (see Table 4).

## 4 DISCUSSION

The purpose of this study was to investigate the effect of habitual RE training on the cerebrovascular response to dynamic RE. We observed no difference in average MCAv_mean_ or zenith‐to‐nadir difference during RE between groups. However, this occurred despite higher average MAP and SBP, and zenith‐to‐nadir MAP difference during RE in the RE‐trained group. Collectively, these data indicate that despite higher within exercise average blood pressures, and greater fluctuations in blood pressure, in the RE‐trained group, MCAv_mean_ responses were not different. These findings align with our hypothesis and suggest that RE‐trained individuals can maintain a more stable MCAv despite more profound changes in MAP.

In the current study we have found no group differences in cardiovascular or cerebrovascular measures at the initial baseline period (pre‐exercise) or between sets. Notably, there was similar ‘drift’ between groups in the baseline periods between sets, for example, MAP was similarly elevated in both groups between sets compared to the initial baseline period prior to exercise (see Table 2). However, MCAv_mean,_ was reduced from the initial baseline period prior to each subsequent exercise bout in both groups. Repetitive exposure to extreme ABP perturbations during high intensity dynamic RE (e.g., ∼80% of 1RM) has been reported to produce unfavourable adaptations within the circulatory system, evidenced by a reduction in central arterial compliance (DeVan et al., 2005; Miyachi, 2013; Miyachi et al., 2004; Okamoto et al., 2009; Palmiere et al., 2018) and increased cerebrovascular resistance at rest compared to sedentary controls (Nakamura & Muraoka, 2018; Thomas et al., 2021), although the latter is not a consistent finding (Corkery et al., 2021). Koch et al. (2005) reported that cerebral autoregulation is temporarily impaired immediately (<90 s) following RE in RE‐trained individuals. In a mixed cohort of RE‐trained and untrained individuals, Smail et al. (2023) reported that transfer function derived gain at 0.10 Hz was increased from baseline 10 min post‐RE but recovered at 45 min. Anterograde shear rate and blood flow can increase in inactive limbs following dynamic RE (Thomas et al., 2020) with an increase in blood flow turbulence and intensity‐dependent increase in endothelial shear stress in the common carotid artery during dynamic RE (Montalvo et al., 2022). These data indicate that dynamic RE produces profound increases in shear rate and endothelial shear stress, accompanied with sinusoidal changes in cerebral blood flow and perfusion pressure that may acutely alter cerebrovascular function. Whilst we report no change in baseline MCAv_mean_ values between groups, repeated exposure to such conditions induced by habitual RE may alter the within‐RE cerebrovascular responses.

To more accurately categorise the nature of the within‐RE haemodynamic profile we analysed the averaged data, the zenith and nadir, and zenith‐to‐nadir difference for both MAP and MCAv_mean_. We demonstrate that despite more profound increases in average MAP during exercise, and greater perturbations in blood pressure (e.g., zenith‐to‐nadir difference for MAP) for the RE‐trained group, average and zenith‐to‐nadir difference for MCAv_mean_ was not different (Figure 3 and Table 4). Although we did not directly assess cerebral autoregulation in the current study, we have previously compared cerebral autoregulation between RE‐trained, endurance trained and healthy sedentary, with the RE‐trained group demonstrating a trend towards a lower transfer function phase during forced oscillations in blood pressure (Perry et al. (2019). Hysteresis refers to the asymmetric cerebral autoregulatory response, with more effective cerebral autoregulatory buffering capacity during hypertensive challenges compared to hypotensive insults (Brassard et al., 2017). Roy et al. (2022) reported that RE‐trained individuals did not exhibit hysteresis during 0.10 Hz repeated squat–stands, but the asymmetric autoregulatory responses persisted in sedentary and endurance trained individuals at this frequency. As cerebral autoregulation was not assessed in the current study, and the frequency of blood pressure fluctuations produced were faster (0.25 Hz) than those analysed by Roy et al. (2022), these data cannot be used to indicate the modification or absence of hysteresis. Additionally, rhythmic handgrip exercise has been postulated to produce a sympathetically mediated vasoconstriction of the MCA (Verbree et al., 2017). Following ganglionic blockade, a substantially greater rise in MCAv was observed during the rapid increase in MAP during phase IV of the Valsalva manoeuvre, indicating the presence of autonomic vasoconstriction during rapid increases in perfusion pressure (Zhang et al., 2004), like those experienced during RE. Thus, more widespread cerebral vasoconstriction cannot be excluded, which may act to prevent hyperperfusion when cerebral perfusion pressure is elevated during RE. Further research is required to assess the effect of RE on hysteresis and regulation of CBF by the sympathetic nervous system.

Thomas et al. (2021) assessed the effects of 12 weeks of RE and endurance training on cerebrovascular haemodynamics using a randomised crossover design. Whilst the cerebrovascular responses to RE were not recorded, the authors report that following 12 weeks of RE training larger increases in MAP were apparent during incremental cycling exercise with a concomitant lower MCAv response compared to before the exercise intervention. Additionally, indices of cerebrovascular resistance in the MCA, posterior cerebral artery and internal carotid artery (ICA) were all increased following 12 weeks of RE training, whilst cerebral autoregulation during spontaneous oscillations in blood pressure was unchanged. Our findings largely corroborate those of Thomas et al. (2021) as the current study indicates that compared to the untrained group (healthy sedentary) the RE‐trained group demonstrated a similar within MCAv during RE despite greater average and magnitude of change in MAP Collectively, these data indicate that cerebral autoregulation may be altered by habitual RE with the adaptations only elucidated during exercise, where blood pressure oscillations are rapid and forced as previously suggested (Perry & Lucas, 2021).

Few studies have investigated the cerebrovascular response to dynamic RE. Studies investigating the acute cerebrovascular response to RE have produced equivocal results. When considering the average MCAv_mean_ during RE, an increase (Moralez et al., 2012; Romero & Cooke, 2007), decrease (Dickerman et al., 2000) and no change (Edwards et al. (2002) has been reported. Furthermore, the average peak MCAv values, (the zenith MCAv_mean_ in the current study) was reported to increase from baseline, with the increase independent of exercise intensity, although this is likely due to recruitment of the Valsalva manoeuvre (Perry et al., 2014). Interestingly, the study of Dickerman et al. (2000), which reports a reduction in MCAv during RE, did not report the partial pressure of arterial carbon dioxide or an appropriate proxy (e.g., $P_{\mathrm{ETC}O_{2}}$). The reporting of such data is critical for interpretation as the carbon dioxide content of the arterial blood is the most potent regulator of cerebral blood flow at rest and during exercise as evidenced by a reduction in within‐RE MCAv when lifters hyperventilate prior to exercise onset (Romero & Cooke, 2007). In the current study the gradual reduction in averaged MCAv_mean_ across the sets in both groups (Figure 3) appears to be driven by a declining $P_{\mathrm{ETC}O_{2}}$. Whilst there were no differences between groups, similar reductions in $P_{\mathrm{ETC}O_{2}}$ for both groups across the exercise sets were observed (e.g., set effect), with $P_{\mathrm{ETC}O_{2}}$ lowest in set 3 and 4 (see Table 3). Even small reductions in $P_{\mathrm{ETC}O_{2}}$ could underpin the observed decrease with a 2%–3% reduction in MCAv observed for every mm Hg decrease in $P_{\mathrm{ETC}O_{2}}$ at rest (Brugniaux et al., 2007). Furthermore, although CO_2_ reactivity was not measured, cerebrovascular reactivity to CO_2_ appears to not be altered by habitual RE training when compared to healthy sedentary individuals and aerobically trained individuals (Corkery et al., 2021). The limited research on cerebrovascular responses to dynamic RE has yielded conflicting results, which highlights the importance of accounting for a potent regulator like CO_2_.

This is the first study to our knowledge that assessed the zenith and nadir of MCAv and MAP measures during RE. Dynamic RE causes sinusoidal fluctuations in MAP which are mirrored by MCAv. Previous studies reporting average MCAv and MAP likely oversimplify the within‐RE haemodynamic responses. As the magnitude and rate of change in MAP determine the MCAv response to acute perturbation in blood pressure (Tzeng et al., 2011), the current findings suggest that repetitive RE may subtly modify cerebrovascular function as resistance‐trained individuals exhibit similar fluctuations in MCAv to untrained individuals during dynamic RE despite larger fluctuations in blood pressure. We have previously reported (Perry et al., 2014) that following RE there is a selective decrease in DMCAv. Though there was a training by set interaction for within exercise DMCAv in the current study, *post hoc* tests revealed no differences. Future studies that investigate the haemodynamic responses to RE should consider measuring zenith and nadir MCAv_mean_ and MAP to gain further understanding of the sinusoidal pattern and its influence on the cerebrovascular response.

### 4.1 Limitations

Some limitations must be discussed to contextualise the findings herein. Firstly, we used TCD to measure MCAv as a non‐invasive proxy for cerebral blood flow. Whilst the TCD provides dynamic and continuous measurements, the use of MCAv as a proxy for cerebral blood flow is dependent on a constant diameter of the MCA (Ainslie & Hoiland, 2014). Verbree et al. (2014) observed that a reduction in $P_{\mathrm{ETC}O_{2}}$ of 7.5 mmHg (hypocapnia) did not elicit any significant change in MCA diameter. However, Coverdale et al. (2014) found that the relative decrease in CBF during hypocapnia was 7 ± 4% greater than the TCD‐measured change in MCAv, although the mean $P_{\mathrm{ETC}O_{2}}$ during hypocapnia was ∼23 torr, which is a considerably larger hypocapnic stimulus than the current study. We implemented paced breathing during RE in the current study, which produced a small decrease (∼1–2 mmHg) in $P_{\mathrm{ETC}O_{2}}$. Thus, it is unlikely that the mild hypocapnia alone produced a change in MCA diameter. However, using high‐resolution magnetic resonance imaging, Verbree et al. (2017) reported that simple handgrip exercise produces a 2% decrease in MCA cross‐section which was suggested to reflect sympathetic vasoconstriction. It is therefore possible that the RE utilised in the current study produced a constriction of the MCA. As such, the within‐exercise findings of the current study must be interpreted with caution.

The current study did not measure blood flow in the ICA or the external carotid artery (ECA). Hirasawa et al. (2016) reported increases in ECA blood flow during low intensity (30% of maximum voluntary contraction) static RE. However, the current study used dynamic RE and a higher intensity (60% of 1RM). The dynamic nature of RE raises methodological challenges when measuring blood flow, namely movement of the participant during exercise and the sinusoidal fluctuations in blood pressure and flow. These considerations precluded the measurement of ICA or ECA blood flow in the current experiment but does mean that we cannot exclude the occurrence of an extracranial shunt. However, as the Valsalva manoeuvre was not utilised in the current experiment, and the within‐RE increases in MAP were modest, shunting of blood to the ECA may have been limited.

The current study included both female and male participants. Cardiovascular differences between females and males have been identified during static handgrip exercise, with male participants showing a greater exercise pressor response with a larger increase in blood pressure during exercise (Ettinger et al., 1996; Matthews & Stoney, 1988; Simoes et al., 2013). However, recent studies investigating sex differences in haemodynamic responses to dynamic exercise have found that when body surface area and composition (Bassareo & Crisafulli, 2020), as well as maximal voluntary contraction (Notay et al., 2018), body size and strength measurements are similar (no statistical differences), the differences in exercise pressor reflex are small or absent (Tharpe et al., 2023). The anthropometric measures of the participants in both groups in the current study were not different, and furthermore there were no baseline differences in cardiovascular measures. A systematic review and meta‐analysis examining cerebrovascular function across the menstrual cycle found that during the high hormone phase, females exhibited higher PI and resistance and lower cerebral blood flow and cerebral autoregulation compared to the low hormone phase (Skinner et al., 2021). However, some studies included in the meta‐analysis investigating the changes in PI and resistance measured ICA blood flow instead of MCAv. Abidi et al. (2017) found that MAP was greater during the high hormone phase versus the lower hormone phase during the Valsalva manoeuvre. However, the authors did not provide baseline cerebrovascular measures between males and females and phase, only the cerebrovascular measures in response to a stressor, and therefore it was difficult to distinguish if there were sex difference at baseline at the different stages of the menstrual cycle. Favre and Serrador (2019) found that cerebral autoregulation was lower during squat‐to‐stand manoeuvres; however, the authors used saliva to measure their oestradiol concentrations, and did not find any significant difference in salivary oestradiol across the menstrual cycle, indicating that blood hormone concentrations are a more accurate form of measure for hormone concentrations. Favre and Serrador (2019) did provide baseline measures for cerebrovascular measures across the menstrual cycle, and between males and females, with no differences apparent. The authors also found that there was no difference in cerebral autoregulation during both repeated squat‐to‐stand and sit‐to‐stand manoeuvres between males and females. Skinner et al. (2021) and Abidi et al. (2017) highlighted the importance of measuring cerebral blood flow during the same phase of the menstrual cycle for consistency as high hormone and low hormone levels can give rise to different blood pressure and HR responses to acute stressors. However, Korad et al. (2022) and Favre and Serrador (2019) found that menstrual cycle phase does not alter cerebrovascular responses during stressors that alter MAP acutely.

### 4.2 Conclusion

The current findings indicate that despite RE‐trained individuals demonstrating greater fluctuations in blood pressure during dynamic lower body RE, MCAv_mean_ was not different versus their untrained counterparts. Therefore, it is possible that engaging in habitual resistance training may produce functional vascular adaptations that maintain cerebral blood flow during RE despite greater blood pressure. Future studies should consider the sinusoidal nature of blood pressure during RE to better characterise the cerebrovascular response during dynamic RE.

## AUTHOR CONTRIBUTIONS

Stephanie Korad, Toby Mündel and Blake G. Perry, contributed to conceptualisation and design of the research. Stephanie Korad and Blake G. Perry were responsible for data collection. Stephanie Korad, Toby Mündel and Blake G. Perry were responsible for data analysis, interpretation and drafting of the article. All authors have read and reviewed the article and provided critical feedback. All authors have approved the final version of this manuscript and agree to be accountable for all aspects of the work in ensuring that questions related to the accuracy or integrity of any part of the work are appropriately investigated and resolved. All persons designated as authors qualify for authorship, and all those who qualify for authorship are listed.

## CONFLICT OF INTEREST

None declared.

## Data Availability

The data that support the findings of this study are available from the corresponding author upon reasonable request.

## References

[eph13587-bib-0001] Aaslid, R. , Lindegaard, K. F. , Sorteberg, W. , & Nornes, H. (1989). Cerebral autoregulation dynamics in humans. Stroke, 20(1), 45–52. 2492126 10.1161/01.str.20.1.45

[eph13587-bib-0002] Abidi, S. , Nili, M. , Serna, S. , Kim, S. , Hazlett, C. , & Edgell, H. (2017). Influence of sex, menstrual cycle, and oral contraceptives on cerebrovascular resistance and cardiorespiratory function during Valsalva or standing. Journal of Applied Physiology, 123(2), 375–386. 28522756 10.1152/japplphysiol.00035.2017 PMC5583611

[eph13587-bib-0003] Ainslie, P. N. , & Hoiland, R. L. (2014). Transcranial doppler ultrasound: Valid, invalid, or both (Vol. 117, pp. 1081–1083). American Physiological Society Bethesda. 25257879 10.1152/japplphysiol.00854.2014

[eph13587-bib-0004] Bassareo, P. P. , & Crisafulli, A. (2020). Gender differences in hemodynamic regulation and cardiovascular adaptations to dynamic exercise. Current Cardiology Reviews, 16(1), 65–72. 30907327 10.2174/1573403X15666190321141856 PMC7393595

[eph13587-bib-0005] Bathala, L. , Mehndiratta, M. M. , & Sharma, V. K. (2013). Transcranial doppler: Technique and common findings (Part 1). Annals of Indian Academy of Neurology, 16(2), 174–179. 23956559 10.4103/0972-2327.112460 PMC3724069

[eph13587-bib-0006] Brassard, P. , Ferland‐Dutil, H. , Smirl, J. D. , Paquette, M. , Le Blanc, O. , Malenfant, S. , & Ainslie, P. N. (2017). Evidence for hysteresis in the cerebral pressure‐flow relationship in healthy men. American Journal of Physiology. Heart and Circulatory Physiology, 312(4), H701–H704. 28130339 10.1152/ajpheart.00790.2016

[eph13587-bib-0007] Braz, I. D. , Scott, C. , Simpson, L. L. , Springham, E. L. , Tan, B. W. L. , Balanos, G. M. , & Fisher, J. P. (2014). Influence of muscle metaboreceptor stimulation on middle cerebral artery blood velocity in humans. Experimental Physiology, 99(11), 1478–1487. 25217497 10.1113/expphysiol.2014.081687

[eph13587-bib-0008] Brugniaux, J. V. , Hodges, A. N. H. , Hanly, P. J. , & Poulin, M. J. (2007). Cerebrovascular responses to altitude. Respiratory Physiology & Neurobiology, 158(2‐3), 212–223. 17544954 10.1016/j.resp.2007.04.008

[eph13587-bib-0009] Brzycki, M. (1993). Strength Testing—Predicting a One‐Rep Max from Reps‐to‐Fatigue. Journal of Physical Education, Recreation & Dance, 64(1), 88–90.

[eph13587-bib-0064] Cohen, J. (2013). Statistical power analysis for the behavioral sciences. Routledge.

[eph13587-bib-0010] Compton, D. , Hill, P. M. , & Sinclair, J. D. (1973). Weight‐lifters' blackout. The Lancet, 302(7840), 1234–1237. 10.1016/s0140-6736(73)90974-4 4128562

[eph13587-bib-0011] Corkery, A. T. , Howery, A. J. , Miller, K. B. , & Barnes, J. N. (2021). Influence of habitual aerobic and resistance exercise on cerebrovascular reactivity in healthy young adults. Journal of Applied Physiology, 130(6), 1928–1935. 33886384 10.1152/japplphysiol.00823.2020 PMC8285606

[eph13587-bib-0012] Coverdale, N. S. , Gati, J. S. , Opalevych, O. , Perrotta, A. , & Shoemaker, J. K. (2014). Cerebral blood flow velocity underestimates cerebral blood flow during modest hypercapnia and hypocapnia. Journal of Applied Physiology, 117(10), 1090–1096. 25012027 10.1152/japplphysiol.00285.2014

[eph13587-bib-0065] Dickerman, R. D. , McConathy, W. J. , Smith, G. H. , East, J. W. , & Rudder, L. (2000). Middle cerebral artery blood flow velocity in elite power athletes during maximal weight‐lifting. Neurological Research, 22(4), 337–340. 10874679 10.1080/01616412.2000.11740679

[eph13587-bib-0013] Deschenes, M. R. , & Kraemer, W. J. (2002). Performance and physiologic adaptations to resistance training. American Journal of Physical Medicine & Rehabilitation, 81(11), S3–S16. 12409807 10.1097/00002060-200211001-00003

[eph13587-bib-0014] DeVan, A. E. , Anton, M. M. , Cook, J. N. , Neidre, D. B. , Cortez‐Cooper, M. Y. , & Tanaka, H. (2005). Acute effects of resistance exercise on arterial compliance. Journal of Applied Physiology, 98(6), 2287–2291. 15718412 10.1152/japplphysiol.00002.2005

[eph13587-bib-0015] Edwards, M. R. , Martin, D. H. , & Hughson, R. L. (2002). Cerebral hemodynamics and resistance exercise. Medicine and Science in Sports and Exercise, 34(7), 1207–1211. 12131264 10.1097/00005768-200207000-00024

[eph13587-bib-0016] Ettinger, S. M. , Silber, D. H. , Collins, B. G. , Gray, K. S. , Sutliff, G. , Whisler, S. K. , McClain, J. M. , Smith, M. B. , Yang, Q. X. , & Sinoway, L. I. (1996). Influences of gender on sympathetic nerve responses to static exercise. Journal of Applied Physiology, 80(1), 245–251. 8847310 10.1152/jappl.1996.80.1.245

[eph13587-bib-0017] Evans, P. L. , McMillin, S. L. , Weyrauch, L. A. , & Witczak, C. A. (2019). Regulation of skeletal muscle glucose transport and glucose metabolism by exercise training. Nutrients, 11(10), 2432. 31614762 10.3390/nu11102432 PMC6835691

[eph13587-bib-0018] Favre, M. E. , & Serrador, J. M. (2019). Sex differences in cerebral autoregulation are unaffected by menstrual cycle phase in young, healthy women. American Journal of Physiology. Heart and Circulatory Physiology, 316(4), H920–H933. 30707610 10.1152/ajpheart.00474.2018

[eph13587-bib-0063] Gosling, R. G. , & King, D. H. (1974). The role of measurement in peripheral vascular surgery: arterial assessment by Doppler‐shift ultrasound. 10.1177/00359157740676P113 PMC1645777 4850636

[eph13587-bib-0019] Hirasawa, A. I. , Sato, K. , Yoneya, M. , Sadamoto, T. , Bailey, D. M. , & Ogoh, S. (2016). Heterogeneous regulation of brain blood flow during low‐intensity resistance exercise. Medicine & Science in Sports & Exercise, 48(9), 1829–1834. 27054676 10.1249/MSS.0000000000000948

[eph13587-bib-0020] Koch, A. , Ivers, M. , Gehrt, A. , Schnoor, P. , Rump, A. , & Rieckert, H. (2005). Cerebral autoregulation is temporarily disturbed in the early recovery phase after dynamic resistance exercise. Clinical Autonomic Research, 15(2), 83. 15834764 10.1007/s10286-005-0249-8

[eph13587-bib-0021] Korad, S. , Mundel, T. , Fan, J. L. , & Perry, B. G. (2022). Cerebral autoregulation across the menstrual cycle in eumenorrheic women. Physiological Reports, 10(9), e15287. 35524340 10.14814/phy2.15287 PMC9076937

[eph13587-bib-0022] Kraemer, W. J. , Deschenes, M. R. , & Fleck, S. J. (1988). Physiological adaptations to resistance exercise. Sports Medicine, 6(4), 246–256. 3067312 10.2165/00007256-198806040-00006

[eph13587-bib-0023] Libardi, C. A. , Catai, A. M. , Miquelini, M. , Borghi‐Silva, A. , Minatel, V. , Alvarez, I. F. , Milan‐Mattos, J. C. , Roschel, H. , Tricoli, V. , & Ugrinowitsch, C. (2017). Hemodynamic responses to blood flow restriction and resistance exercise to muscular failure. International Journal of Sports Medicine, 38(02), 134–140. 27931053 10.1055/s-0042-115032

[eph13587-bib-0024] Lopez, P. , Taaffe, D. R. , Galvão, D. A. , Newton, R. U. , Nonemacher, E. R. , Wendt, V. M. , Bassanesi, R. N. , Turella, D. J. P. , & Rech, A. (2022). Resistance training effectiveness on body composition and body weight outcomes in individuals with overweight and obesity across the lifespan: A systematic review and meta‐analysis. Obesity Reviews, 23(5), e13428. 35191588 10.1111/obr.13428 PMC9285060

[eph13587-bib-0025] MacDougall, J. D. , Tuxen, D. , Sale, D. G. , Moroz, J. R. , & Sutton, J. R. (1985). Arterial blood pressure response to heavy resistance exercise. Journal of Applied Physiology, 58(3), 785–790. 3980383 10.1152/jappl.1985.58.3.785

[eph13587-bib-0026] Mattace‐Raso, F. U. S. , van der Cammen, T. J. M. , Hofman, A. , van Popele, N. M. , Bos, M. L. , Schalekamp, M. A. D. H. , Asmar, R. , Reneman, R. S. , Hoeks, A. P. G. , Breteler, M. M. B. , & Witteman, J. C. M. (2006). Arterial stiffness and risk of coronary heart disease and stroke. Circulation, 113(5), 657–663. 16461838 10.1161/CIRCULATIONAHA.105.555235

[eph13587-bib-0027] Matthews, K. A. , & Stoney, C. M. (1988). Influences of sex and age on cardiovascular responses during stress. Psychosomatic Medicine, 50(1), 46–56. 3344302 10.1097/00006842-198801000-00006

[eph13587-bib-0028] Mitchell, G. F. , Hwang, S.‐J. , Vasan, R. S. , Larson, M. G. , Pencina, M. J. , Hamburg, N. M. , Vita, J. A. , Levy, D. , & Benjamin, E. J. (2010). Arterial stiffness and cardiovascular events. Circulation, 121(4), 505–511. 20083680 10.1161/CIRCULATIONAHA.109.886655 PMC2836717

[eph13587-bib-0029] Miyachi, M. (2013). Effects of resistance training on arterial stiffness: A meta‐analysis. British journal of sports medicine, 47(6), 393–396. 22267567 10.1136/bjsports-2012-090488

[eph13587-bib-0030] Miyachi, M. , Kawano, H. , Sugawara, J. , Takahashi, K. , Hayashi, K. , Yamazaki, K. , Tabata, I. , & Tanaka, H. (2004). Unfavorable effects of resistance training on central arterial compliance. Circulation, 110(18), 2858–2863. 15492301 10.1161/01.CIR.0000146380.08401.99

[eph13587-bib-0031] Montalvo, S. , Gomez, M. , Lozano, A. , Arias, S. , Rodriguez, L. , Morales‐Acuna, F. , & Gurovich, A. N. (2022). Differences in blood flow patterns and endothelial shear stress at the carotid artery using different exercise modalities and intensities. Frontiers in Physiology, 13, 922. 10.3389/fphys.2022.857816 PMC9127153 35620608

[eph13587-bib-0032] Moralez, G. , Romero, S. A. , Rickards, C. A. , Ryan, K. L. , Convertino, V. A. , & Cooke, W. H. (2012). Effects of dehydration on cerebrovascular control during standing after heavy resistance exercise. Journal of Applied Physiology, 112(11), 1875–1883. 22461441 10.1152/japplphysiol.01217.2011

[eph13587-bib-0033] Nakamura, N. , & Muraoka, I. (2018). Resistance training augments cerebral blood flow pulsatility: Cross‐sectional study. American Journal of Hypertension, 31(7), 811–817. 29506139 10.1093/ajh/hpy034

[eph13587-bib-0034] Notay, K. , Lee, J. B. , Incognito, A. V. , Seed, J. D. , Arthurs, A. A. , & Millar, P. J. (2018). Muscle strength influences pressor responses to static handgrip in men and women. Medicine and Science in Sports and Exercise, 50(4), 778–784. 29135658 10.1249/MSS.0000000000001485

[eph13587-bib-0035] O'Connor, P. J. , Herring, M. P. , & Caravalho, A. (2010). Mental health benefits of strength training in adults. American Journal of Lifestyle Medicine, 4(5), 377–396.

[eph13587-bib-0036] Okamoto, T. , Masuhara, M. , & Ikuta, K. (2009). Upper but not lower limb resistance training increases arterial stiffness in humans. European Journal of Applied Physiology, 107, 127–134. 19533164 10.1007/s00421-009-1110-x

[eph13587-bib-0037] Palmiere, S. , Wade, M. , DeBlois, J. P. , Lefferts, W. K. , & Heffernan, K. S. (2018). Aortic stiffness, central pulse pressure and cognitive function following acute resistance exercise. European Journal of Applied Physiology, 118, 2203–2211. 30056548 10.1007/s00421-018-3948-2

[eph13587-bib-0038] Perry, B. G. , Bear, T. L. K. , Lucas, S. J. E. , & Mündel, T. (2016). Mild dehydration modifies the cerebrovascular response to the cold pressor test. Experimental Physiology, 101(1), 135–142. 26374269 10.1113/EP085449

[eph13587-bib-0039] Perry, B. G. , Cotter, J. D. , Korad, S. , Lark, S. , Labrecque, L. , Brassard, P. , Paquette, M. , Le Blanc, O. , & Lucas, S. J. E. (2019). Implications of habitual endurance and resistance exercise for dynamic cerebral autoregulation. Experimental Physiology, 104(12), 1780–1789. 31549452 10.1113/EP087675

[eph13587-bib-0040] Perry, B. G. , De Hamel, T. , Thomas, K. N. , Wilson, L. C. , Gibbons, T. D. , & Cotter, J. D. (2020). Cerebrovascular haemodynamics during isometric resistance exercise with and without the Valsalva manoeuvre. European Journal of Applied Physiology, 120(2), 467–479. 31912226 10.1007/s00421-019-04291-7

[eph13587-bib-0041] Perry, B. G. , & Lucas, S. J. E. (2021). The acute cardiorespiratory and cerebrovascular response to resistance exercise. Sports Medicine‐Open, 7(1), 1–19. 34046740 10.1186/s40798-021-00314-w PMC8160070

[eph13587-bib-0042] Perry, B. G. , Schlader, Z. J. , Barnes, M. J. , Cochrane, D. J. , Lucas, S. J. E. , & MüNdel, T. (2014). Hemodynamic response to upright resistance exercise: Effect of load and repetition. Medicine & Science in Sports & Exercise, 46(3), 479–487. 23917471 10.1249/MSS.0b013e3182a7980f

[eph13587-bib-0043] Pott, F. , Knudsen, L. , Nowak, M. , Nielsen, H. B. , Hanel, B. , & Secher, N. H. (1997). Middle cerebral artery blood velocity during rowing. Acta Physiologica Scandinavica, 160(3), 251–255. 9246388 10.1046/j.1365-201X.1997.00144.x

[eph13587-bib-0044] Romero, S. A. , & Cooke, W. H. (2007). Hyperventilation before resistance exercise: Cerebral hemodynamics and orthostasis. Medicine & Science in Sports & Exercise, 39(8), 1302–1307. 17762363 10.1249/mss.0b013e3180653636

[eph13587-bib-0045] Roy, M. A. , Labrecque, L. , Perry, B. G. , Korad, S. , Smirl, J. D. , & Brassard, P. (2022). Directional sensitivity of the cerebral pressure‐flow relationship in young healthy individuals trained in endurance and resistance exercise. Experimental Physiology, 107(4), 299–311. 35213765 10.1113/EP090159

[eph13587-bib-0046] Seguin, R. , & Nelson, M. E. (2003). The benefits of strength training for older adults. American Journal of Preventive Medicine, 25, (3, Supplement 2), 141–149. 14552938 10.1016/s0749-3797(03)00177-6

[eph13587-bib-0047] Simoes, G. M. S. , Campagnaro, B. P. , Tonini, C. L. , Meyrelles, S. S. , Kuniyoshi, F. H. S. , & Vasquez, E. C. (2013). Hemodynamic reactivity to laboratory stressors in healthy subjects: Influence of gender and family history of cardiovascular diseases. International Journal of Medical Sciences, 10(7), 848. 23794949 10.7150/ijms.5967 PMC3689876

[eph13587-bib-0048] Skinner, B. D. , Davies, R. J. , Weaver, S. R. , Cable, N. T. , Lucas, S. J. E. , & Lucas, R. A. I. (2021). A systematic review and meta‐analysis examining whether changing ovarian sex steroid hormone levels influence cerebrovascular function. Frontiers in Physiology, 12, 687591. 34220552 10.3389/fphys.2021.687591 PMC8248489

[eph13587-bib-0049] Smail, O. J. , Clarke, D. J. , Al‐Alem, Q. , Wallis, W. , Barker, A. R. , Smirl, J. D. , & Bond, B. (2023). Resistance exercise acutely elevates dynamic cerebral autoregulation gain. Physiological Reports, 11(8), e15676. 37100594 10.14814/phy2.15676 PMC10132945

[eph13587-bib-0050] Strasser, B. , & Schobersberger, W. (2011). Evidence for resistance training as a treatment therapy in obesity. Journal of Obesity, 2011, 482564. 20847892 10.1155/2011/482564 PMC2931407

[eph13587-bib-0051] Tharpe, M. A. , Linder, B. A. , Babcock, M. C. , Watso, J. C. , Pollin, K. U. , Hutchison, Z. J. , Barnett, A. M. , Culver, M. N. , Kavazis, A. N. , & Brian, M. S. (2023). Adjusting for muscle strength and body size attenuates sex differences in the exercise pressor reflex in young adults. American Journal of Physiology. Heart and Circulatory Physiology, 325(6), H1418–H1429. 37861651 10.1152/ajpheart.00151.2023 PMC10907031

[eph13587-bib-0052] Thomas, H. J. , Marsh, C. E. , Naylor, L. H. , Ainslie, P. N. , Smith, K. J. , Carter, H. H. , & Green, D. J. (2021). Resistance, but not endurance exercise training, induces changes in cerebrovascular function in healthy young subjects. American Journal of Physiology. Heart and Circulatory Physiology, 321(5), H881–H892. 34559581 10.1152/ajpheart.00230.2021

[eph13587-bib-0053] Thomas, K. N. , Kissling, L. S. , Gibbons, T. D. , Akerman, A. P. , van Rij, A. M. , & Cotter, J. D. (2020). The acute effect of resistance exercise on limb blood flow. Experimental Physiology, 105(12), 2099–2109. 33058304 10.1113/EP088743

[eph13587-bib-0054] Tzeng, Y.‐C. , Chan, G. S. H. , Willie, C. K. , & Ainslie, P. N. (2011). Determinants of human cerebral pressure–flow velocity relationships: New insights from vascular modelling and Ca2+ channel blockade. The Journal of Physiology, 589(13), 3263–3274. 21540346 10.1113/jphysiol.2011.206953 PMC3145938

[eph13587-bib-0055] Verbree, J. , Bronzwaer, A. G. T. , van Buchem, M. A. , Daemen, M. , van Lieshout, J. J. , & van Osch, M. J. P. (2017). Middle cerebral artery diameter changes during rhythmic handgrip exercise in humans. Journal of Cerebral Blood Flow & Metabolism, 37(8), 2921–2927. 27837189 10.1177/0271678X16679419 PMC5536799

[eph13587-bib-0056] Verbree, J. , Bronzwaer, A.‐S. G. T. , Ghariq, E. , Versluis, M. J. , Daemen, M. J. A. P. , van Buchem, M. A. , Dahan, A. , van Lieshout, J. J. , & van Osch, M. J. P. (2014). Assessment of middle cerebral artery diameter during hypocapnia and hypercapnia in humans using ultra‐high‐field MRI. Journal of Applied Physiology, 117(10), 1084–1089. 25190741 10.1152/japplphysiol.00651.2014

[eph13587-bib-0057] Willie, C. K. , Colino, F. L. , Bailey, D. M. , Tzeng, Y. C. , Binsted, G. , Jones, L. W. , Haykowsky, M. J. , Bellapart, J. , Ogoh, S. , Smith, K. J. , Smirl, J. D. , Day, T. A. , Lucas, S. J. , Eller, L. K. , & Ainslie, P. N. (2011). Utility of transcranial Doppler ultrasound for the integrative assessment of cerebrovascular function. Journal of Neuroscience Methods, 196(2), 221–237. 21276818 10.1016/j.jneumeth.2011.01.011

[eph13587-bib-0058] Yarrow, J. F. , White, L. J. , McCoy, S. C. , & Borst, S. E. (2010). Training augments resistance exercise induced elevation of circulating brain derived neurotrophic factor (BDNF). Neuroscience Letters, 479(2), 161–165. 20553806 10.1016/j.neulet.2010.05.058

[eph13587-bib-0059] Zhang, R. , Crandall Craig, G. , & Levine Benjamin, D. (2004). Cerebral hemodynamics during the Valsalva maneuver. Stroke, 35(4), 843–847. 14976327 10.1161/01.STR.0000120309.84666.AE

[eph13587-bib-0060] Zhang, R. , Zuckerman, J. H. , Giller, C. A. , & Levine, B. D. (1998). Transfer function analysis of dynamic cerebral autoregulation in humans. American Journal of Physiology. Heart and Circulatory Physiology, 274(1), H233–H241. 10.1152/ajpheart.1998.274.1.h233 9458872

[eph13587-bib-0061] Zhang, R. , Zuckerman, J. H. , Iwasaki, K. , Wilson, T. E. , Crandall, C. G. , & Levine, B. D. (2002). Autonomic neural control of dynamic cerebral autoregulation in humans. Circulation, 106(14), 1814–1820. 12356635 10.1161/01.cir.0000031798.07790.fe

